# Biomonitoring polycyclic aromatic hydrocarbon levels in domestic kitchens using commonly grown culinary herbs

**DOI:** 10.1007/s40201-024-00898-x

**Published:** 2024-04-23

**Authors:** Bettina Eck-Varanka, Katalin Hubai, Nora Kováts, Gábor Teke

**Affiliations:** 1 Centre for Natural Sciences, University of Pannonia, Egyetem Str. 10, 8200 Veszprém, Hungary; 2 ELGOSCAR-2000 Environmental Technology and Water Management Ltd, 8184 Balatonfűzfő, Hungary

**Keywords:** Cooking-related emissions, Polycyclic aromatic hydrocarbons, Indoor air quality, Biomonitoring, Culinary herbs

## Abstract

Cooking is a significant source of polycyclic aromatic hydrocarbon (PAHs) emissions in indoor environments. A one-month biomonitoring study was carried out in previously selected rural Hungarian kitchens to evaluate cooking-related PAHs concentrations in 4 common kitchen vegetables such as basil, parsley, rocket and chives. The study had two mainobjectives: firstly, to follow PAHs accumulation pattern and to find out if this pattern can be associated with different cooking habits. Also, the usefulness of culinary herbs for indoor bioaccumulation studies was assessed. The 2-ring naphthalene was the dominant PAH in the majority of the samples, its concentrations were in the range of 25.4 µg/kg and 274 µg/kg, of 3-ring PAHs the prevalency of phenanthrene was observed, with highest concentration of 62 µg/kg. PAHs accumulation pattern in tested plants clearly indicated differences in cooking methods and cooking oils used in the selected households. Use of lard and animal fats in general resulted in the high concentrations of higher molecular weight (5- and 6-ring) PAHs, while olive oil usage could be associated with the emission of 2- and 3-ring PAHs. Culinary herbs, however, accumulated carcinogenic PAHs such as benzo[a]anthracene (highest concentration 11.9 µg/kg), benzo[b]fluoranthene (highest concentration 13.8 µg/kg) and chrysene (highest concentration 20.1 µg/kg) which might question their safe use.

## Introduction

Indoor air quality has become a crucial issue (WHO 2010), especially considering the fact that people spend around 90% of their time indoors in Europe [18]. Cooking is a major indoor source of polycyclic aromatic hydrocarbons (PAHs) [61]; some authors even argue that it has a significant contribution to urban outdoor PAH concentrations for example via neighbourhood diffusion [13]. PAHs are listed by the EU Directive 2004/107/EC [14] among the contaminants of emerging environmental concern.

Cooking-related emissions differ considerably, depending on cooking practices and methods [59], as well as materials used. Deep-frying is the most common procedure to perapare food [16], as deep-fried items usually have attractive color and texture [5]. However, deep-frying is reportedly generating the highest amount of PAHs as a result of the high oil temperatures used [1]. In the experiments of Yao et al. [60], average oil temperature measured during deep-frying was 203 °C, in comparison to the temperature during frying (139 °C). Oil temperatures determine PAHs emissions in cooking fumes as PAHs move from the oil into the air when heated [35]. An additional problem associated with deep-frying is that vegetable oils are often re-used mainly to reduce costs. During repeated use, nutritional value and safety decrease [38]. Manzoor et al. [32] reported that only 20% of the in-use frying oil and 10% food products were safe for consumption in street vendors in Kashmir.

There are a wide range of studies analysing PAHs emission during different cooking activities and in different environments. Nevertheless, in case of chemical monitoring, sampling is done for a very limited duration, mostly covering the time of the experimental cooking (e.g. Alves et al. [2, 29, 63]. On the contrary, using biomonitors, integrated exposure of pollutants can be assessed for a pre-chosen continous or semi-continous exposure [36]. Plants provide an excellent tool for PAHs biomonitoring as they are exposed to PAHs both from gas-phase air and solid particles suspended in the air [56]. Light molecular weight (LMW) PAHs are characteristic in gas phase due to their volatile nature while higher molecular weight (HMW) compounds are less volatile and occur mostly in the airborne particulate form [11].

Different plant species have been widely used to capture information about the level of PAHs pollution in outdoor environments like urban settings (e.g. Klingberg et al. [26], industrial areas (e.g. Bayouli et al. [4] or agricultural lands (e.g. Capozzi et al. [8]. Although more scarcely, indoor applications have also been documented. Rzepka et al. [43] evaluated genotoxic properties of indoor air applying comet assay on *Scindapsus aureus* (pothos). Nap levels were monitored by the *Tradescantia pallida* cv. Purpurea micronucleus assay in the study of Alves et al. [3]. Transplanted lichens have been the most widely used in indoor studies [6, 7, 39, 40, 49]. However, houseplants such as *Dieffenbachia amoena*, *Dracena marginata*, *Ficus elastica* or *Yucca massengena* were used to monitor emissions from tobacco smoke in the study of Ghoma et al. [17]. Tobacco smoke was also monitored by the moss *Pleurozium schreberi* in the study of Świsłowski et al. [51].

It has become a more and more fashionable custom to grow culinary herbs in kitchens providing easy availability during cooking, also, they might have aesthetic value. Considering culinary herbs, most studies report PAH contamination during processing (e.g. Coleto et al. [12]. However, field studies also support that several herb species can contain significant amount of PAHs [57], depending on the site of cultivation and the species.

Such herbs, however, have not been examined in indoor environments. As such, the main goal of the study was to investigate to what extent they can accumulate cooking-generated PAHs in kitchens. In addition to determining their usefulness for indoor bioaccumulation studies, the other main question in the present study was to find out if pattern of accumulated PAHs can be associated with different cooking habits.

## Materials and methods

### Household selection

3 households were selected sharing some important characteristic features (Table 1.). They have similar size, 2 adults + 2 children. Also, they are situated in small villages, not affected by heavy traffic or any outdoor pollution source. As several studies have shown that infiltration of outdoor air pollutants could be influencing indoor air quality in case of traffic-impacted sites (e.g. Tong et al. [52], this criterion was of crucial importance.

**Table 1:** Description of the surveyed households

	HH1	HH2	HH3
Number of inhabitants	4	4	4
Cooking frequency per day	usually 2	1, very seldom 2 (less than 5%)	usually 2
Material used	lard app. 95% butter app. 5%	lard app. 40% vegetable (sunflower) oil app.55% olive oil app.5%	lard 5% vegetable (sunflower) oil app. 95%,
Cooking method	Boiling 30% Deep-frying 10% Pan-frying 40% Oven 20%	Boiling 35% Deep-frying 25% Pan-frying 15% Oven 25%	Boiling 35% Deep frying 25% Pan frying 10% Oven 30%

### Pot experiment

Commonly grown kitchen vegetables have been selected for the study as follows: *Eruca sativa* Mill. (rocket) (Family Brassicaceae); *Ocimum basilicum* L. cv. Genovese (basil) (Family Lamiaceae); *Petroselinum crispum* var. neapolitanum (Mill.) Fuss (leaf parsley) (Family Apiaceae) and *Allium schoenoprasum* L. (chives) (Family Amaryllidaceae). As such, the test battery involved 3 dicot (*E. sativa*, *O. basilicum*, *P. crispum*) and 1 monocot species (*A. schoenoprasum*). These species can be easily kept indoors, in fact, they are recommended for indoor environments by gastroblogs. Leaf surface absorption is the main transport pathway of PAHs from the air in leafy vegetables [62].

Test plants were purchased from local retainers, and acclimatized in a closed greenhouse for 1 month without possible pollution sources. Also, after purchase, they were planted in uncontaminated commercial soil (pH: 6.8 ± 0.5; N (m/m%): min 0.3; P_2_O_5_ (m/m%): min 0.1; K_2_O (m/m%): min 0.3).

Exposure took one month, between 1 June and 30 June. Summer period was chosen to avoid potential cross-pollution from heating. Also, during summer holidays children mostly lunch at home, which increases cooking frequency. During exposure, plants were provided with enough water to prevent water stress.

After the exposure period healthy, app. 1 month old leaves were selected and cut with pre-washed scissors (using ultra-pure water and ethanol). In each household, composite sample was made of every herb species (app. 20–30 g/herb). Samples were immediately taken to the laboratory, washed, ground and homogenized [53]. Prior to analysis, they were kept in the freezer (− 20 °C).

### Determination of the PAHs content

Detailed description of plant material preparation and analytical procedures are given in Hubai et al. [21]. 10 g of each vegetable sample was grinded followed by extraction with 20 mL n-hexane. To reduce the amount of interferes from plant samples additional solid-phase silica gel and alumina oxide sample cleanup was performed. Prior the extraction 10 mL acetone and 100 µL of 0.01 µg/mL deuterated PAHs surrogate mixture (naphtalene-d8, acenaphthene-d10,phenanthrene-d10, chryzene-d12, benzo(a)pyrene-d12, andperylene-d12, from Restek Corporation, Bellefonte, Penn-sylvania, USA) was added and was concentrated to 1 mL using dry nitrogen stream. Solid phase silica gel and alumina oxide sample clean-up was performed than 100 µl of 0.01 µg/mL Internal standard mixture (2-floro-biphenyl, and p-terphenyl-d14) was added to the clean sample. Analyses based on MSZ (Hungarian Standard) EN 15527:2009 (Characterization of waste. Determination of polycyclic aromatic hydrocarbons (PAH) in waste using gas chromatography mass spectrometry). The measurements were performed by HP-6890 gas chromatograph coupled to an HP-5973 (Agilent Technologies, Palo-Alto, USA) quadrupole mass spectrometer (low-resolution single MS). ZB-Semivolatiles (Phenomenex, Torrance California USA) GC column was used. The head pressure of the column during the analysis was 50PSI. After the injection for 180 s the temperature of the GC oven was 40 °C. After it was heated up to to 80 °C (40 °C/min) for 0.5 min, than it was increased 15 °C/min to reach the final temperature (310 °C). Five different concentrations between 0.5 and5.0 ng/mL were detected to determine the calibration curve.

All data were corrected for the average value of the blanks. The PAHs plant samples RSD ranged from 5.47% (Chrysene) to 17.51% (Naphthalene). The average analyte recovery for plant spiked PAHs samples ranged from 88.3% (Benzo(k)fluoranthene) to 106,4 (Anthracene). In the aerosol filter water extract spiked samples RSD ranged from 2.11% (Benzo(g,h,i)perylene) to 10.74% (Naphthalene). The limit of PAH detection (LOD) in plant samples 0.1 µg/kg, the limit of quantifications (LOQ) was 0.05 µg/kg dry plant material. Analytical determinations were performed by the courtesy of the Laboratory of the ELGOSCAR-2000 Environmental Technology and Water Management Ltd. accredited by the (Hungarian) National Accreditation Authority, registration number NAH-1-1278/2015.

### Statistical analysis

In order to examine compositional differences among samples, principal component analysis (PCA) has been performed which generally reduces the set of variables into two major principle components. PCA has been extensively used to evaluate PAH accumulation pattern in different plant matrices (e.g. Kodnik et al. [8, 27]. Statistical analyses were performed using the RStudio (RStudio Desktop 1.4.1106) program, ggfortify package (https://CRAN.R-project.org/package=ggfortify).

## Results and discussion

### Accumulation pattern

Accumulated PAHs are given in Table 2, grouped by households. Except for parsley grown in HH2, accumulation of PAHs in each household was dominated by the 2-ring naphthalene, regardless of the vegetable species used (Table 2), in concordance with other studies (e.g. Zhu and Wang [47, 65]. Chen et al. [9] measured gaseous and particulate emissions in Chinese restaurants and reported that deep frying produced the highest amount of total gaseous PAHs, with the dominance of naphthalene ranging between 67 and 89%. Jia et al. [23] modelled PAHs accumulation in different leafy vegetables and found that gas-phase absorption contributed to app. 90% of total uptake. Nap typically occurs in the gas phase. Generally, higher temperatures lead to higher gas phase PAHs concentrations while in cold temperature high level of PAHs in the particulate phase can be expected [28]. Nap production, however, seems to be independent from cooking styles: Huang et al. [20] measured PAHs emission during frying, steaming and grilling in Chinese commercial kitchens and found similar Nap emissions.

**Table 2:** Concentration of PAHs in the vegetables, grouped by households

PAH	Basil HH1	Rocket HH1	Parsley HH1	Chives HH1	Basil HH2	Rocket HH2	Parsley HH2	Chives HH2	Basil HH3	Rocket HH3	Parsley HH3	Chives HH3
Nap	86	87.4	274	273	73.5	142	< 0.01	25.4	72.4	144	68.8	85.4
Methy-Nap	34.8	4.5	19.1	9.4	10	14.1	0.92	9.9	10	10.1	10.1	9.2
Me-Nap	24	2.8	13.1	6.6	6.2	8.5	7.8	5.8	7.12	7.1	7.2	0.72
Acy	19	2.9	7.2	2.3	1.8	11.4	3	1.7	6.2	5	1	2.1
Ace	1.42	2.66	3	1.5	1.5	2.3	1.9	1	2.1	1	1.2	1.1
Flu	3.14	9.3	17.1	6.3	6.6	11.5	7.6	10.4	6	5.2	0.4	0.4
Phe	1.24	1.16	62	45	24.7	28.9	2.4	4.7	32.1	38.7	28.2	32.2
Ant	1.4	7.8	2.9	3.1	2.4	4.7	0.55	2.5	5	5.4	3.4	4.5
Flt	11.4	8.4	23	12.7	22.5	3.7	20.3	17.6	54.2	2.7	12.2	20
Pyr	8.86	11.2	15.8	7.1	14.5	2.1	11.5	12	3.4	1.7	6.2	10.1
B(a)A	7.06	7.7	7.4	4.9	9.8	11.9	9	8.5	1.8	0.8	4.2	5.05
Cry	16.5	8.04	14.1	6	14.1	20.1	10.2	12.1	2.9	0.92	4	0.62
B(b)F	1.12	5.9	13.5	4.4	13.8	13.2	8.4	1.29	0.28	1.1	9.2	10.1
B(k)F	8.5	4.00	6.7	2.6	7	8.9	4.4	0.6	19	0.6	3.2	0.5
B(e)P	< 0.01	< 0.01	6.74	3	8.2	8.8	4.4	< 0.01	< 0.01	< 0.01	< 0.01	< 0.01
B(a)P	< 0.01	< 0.01	5.7	1.6	3.7	3.1	3.1	< 0.01	< 0.01	< 0.01	< 0.01	< 0.01
D(a.h)A	< 0.01	< 0.01	< 0.01	< 0.01	6.6	6.1	5.6	< 0.01	< 0.01	< 0.01	< 0.01	< 0.01
Ind	< 0.01	< 0.01	< 0.01	< 0.01	3.5	< 0.01	1.6	< 0.01	< 0.01	< 0.01	< 0.01	< 0.01
B(g.h.i)P	< 0.01	< 0.01	< 0.01	< 0.01	7.6	< 0.01	6.2	< 0.01	< 0.01	< 0.01	< 0.01	< 0.01
Total	224.4	163.8	506.9	391.4	238	301.3	108.87	113.49	222	224	159	182

Of 3-ring PAHs, Phen was found in the highest concentrations in the majority of the samples, reaching as much as 62 µg/kg (parsley, HH1). Masuda et al. [33] measured 12 PAHs in cooking exhaust gas, phenanthrene (2100 ng m^− 3^), fluorene (630 ng m^− 3^), and anthracene (200 ng m^− 3^) were detected at the highest concentrations. Sun et al. [50] also reported the dominance of Phen when emissions in Sichuan style restaurant were measured. Sichuan cuisine is generally characterised by quick frying, high-temperature cooking and large oil consumption, which favours to PAHs production. Phen was however measured at high concentrations when emissions from water-based cooking activities were studied in Chinese kitchens [64].

Considering 4-ring PAHs, Flt and Pyr have been reported as being abundant/dominant in cooking-related emissions [50]. These two PAHs were detected in the study of Chen et al. [9] in high concentrations in Chinese-style restaurants characterised by stir-frying and deep-frying while much lower concentrations were found in western-style ones. In our samples, however, no clear pattern was shown. Accumulation of Flt seems to be depending on the vegetable tested: in general, high concentrations were found in basil, parsley and chives but definitely lower accumulation was found in case of rocket. Even less clear tendency was found for Pyr. Cry, however, was found in some samples in higher concentrations than reported by the literature: in HH2 in the range of 10.2 (parsley) and 20.1 (rocket) and also reaching high values in HH1, 16.5 µg/kg in basil and 14.1 µg/kg in parsley.

The 6-ring PAHs, Ind and B(g,h,i.)p were detected only in HH2 (Ind: 3.5 µg/kg in basil and 1.61 µg/kg in parsley, while B(g,h,i.)p 7.6 µg/kg in basil and 6.2 µg/kg in parsley). Occurrence of these PAHs is quite ambiguous in different studies: Zhao et al. [64] found considerably lower concentrations of these PAHs in deep-frying generated emissions in comparison to pan-frying. On the contrary, in the study of Wu et al. [58] these PAHs were detected exclusively in street cart areas, characterised by deep-frying. Similarly, in the above mentioned study of Chen et al. [9], these compounds were found characteristic of Chinese style cooking. These studies alone, however, cannot explain the lack of these compounds in samples collected in HH3, as this household also used deep frying in app. 25% of meal preparation.

**Fig. 1:**
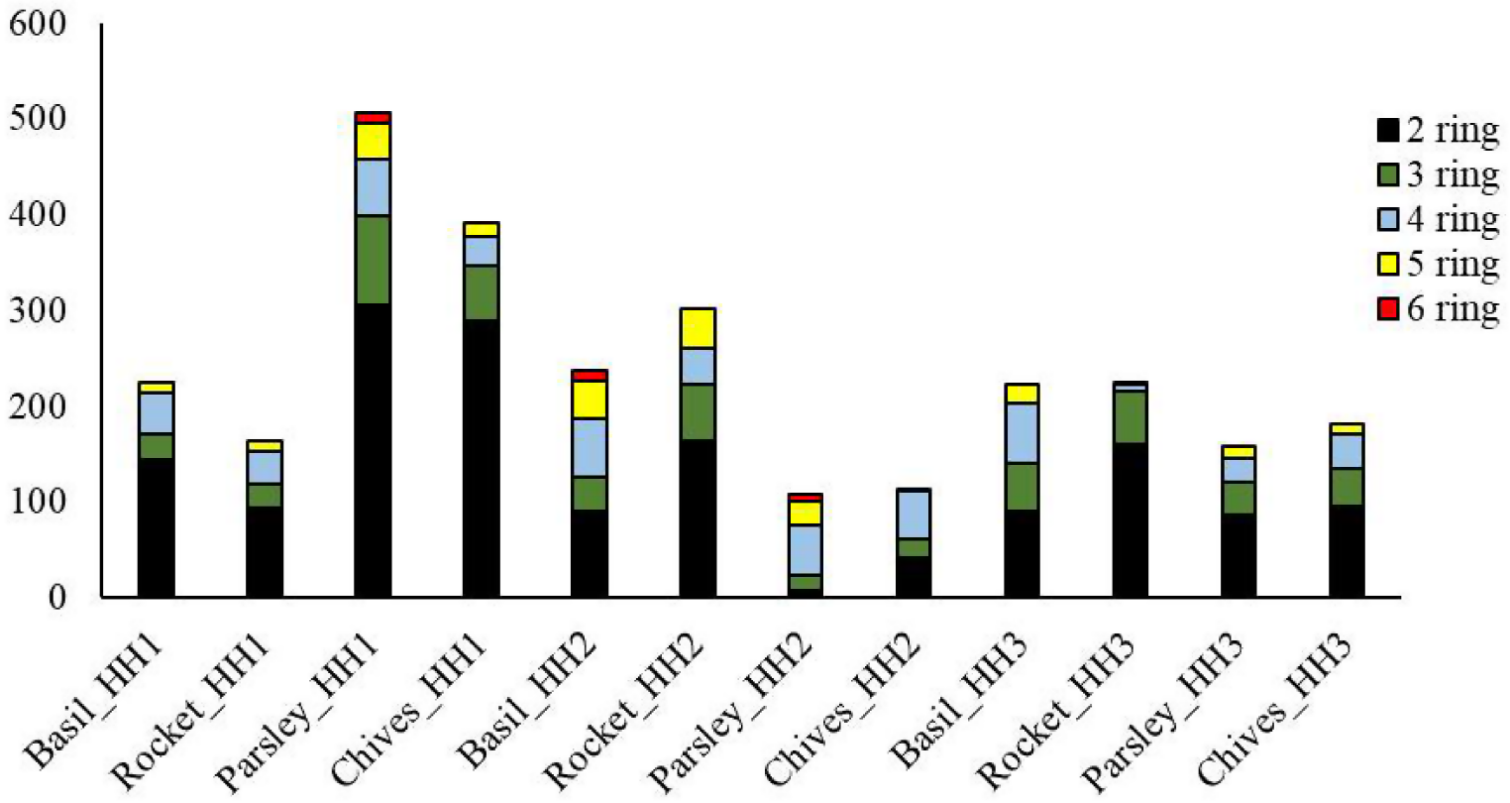
PAHs isomers in the vegetable samples grouped by households

More clear differences can be seen between households when ratio of different molecular weight PAHs is analysed. Figure 1 shows the distribution pattern of PAHs in vegetables/households grouped by the number of rings.

Characteristic differences between the households are represented by the distribution of HMW PAHs. The differences are especially clear when 6-ring PAHs are addressed: their concentration in HH2 was 11.1 in basil and 7.8 in parsley, and 10.4 in parsley in HH1. On the contrary, in HH1 no 6-ring PAHs was detected. HH2 uses animal fat (lard) in app. 40% of its cooking activities and HH1 relies exclusively on the use of animal fats: lard app. 95% and butter app. 5%. The use of lard is negligible in HH3, 5%. Lard is extensively used in Hungary, Rurik and Antal [42] reported that lard was used by 44% of subjects when cooking habits of elderly people was studied. Literature studies concerning emissions generated by animal fat usage as cooking material are extremely rare. Zhu and Wang [65] compared PAHs content of lard and vegetable oil fumes and found that lard fumes released more PAHs when heated at the same temperature. Jing et at. (2022) reported higher PAHs emission and resulting incidence of cancers when animal fat was used, in comparison to vegetable oil use.

More studies have dealt with emissions generated during usage of raw materials with high animal fat content. Li et al. [31] found that fat contents in raw materials can also be an important factor influencing PAHs emission. Rogge et al. [41] demonstrated that PAHs emissions increased with increasing fat content of meat prepared. Of HMW PAHs, B(g,h,i.)p was detected in high concentrations when hamburgers with high fat content were grilled [34]. Alves et al. [2] compared PAHs emissions during cooking different typical Latin meals such as stuffed chicken, fried mackerel, fried and grilled pork. Emissions generated during preparation of grilled pork contained HMW PAHs in high concentrations, including Ind, D(a,h)a and B(g,h,i,)p.

Fats and proteins are cracked under high-temperature conditions (200 ◦C and above), producing first low molecular weight rings. The process finally results in the formation of high molecular weight PAHs (reviewed by Zhu et al. [66]. It is depending on the temperature: Li and Wu [30] demonstrated that total concentrations of selected PAHs in Chinese home-made sausage baked at 220 ◦C were significantly higher than that in the sausage baked at 180 ◦C. The amount of PAHs produced during the pyrolysis of fats increases with higher fat content (reviewed by Neves et al. [37].

**Fig. 2:**
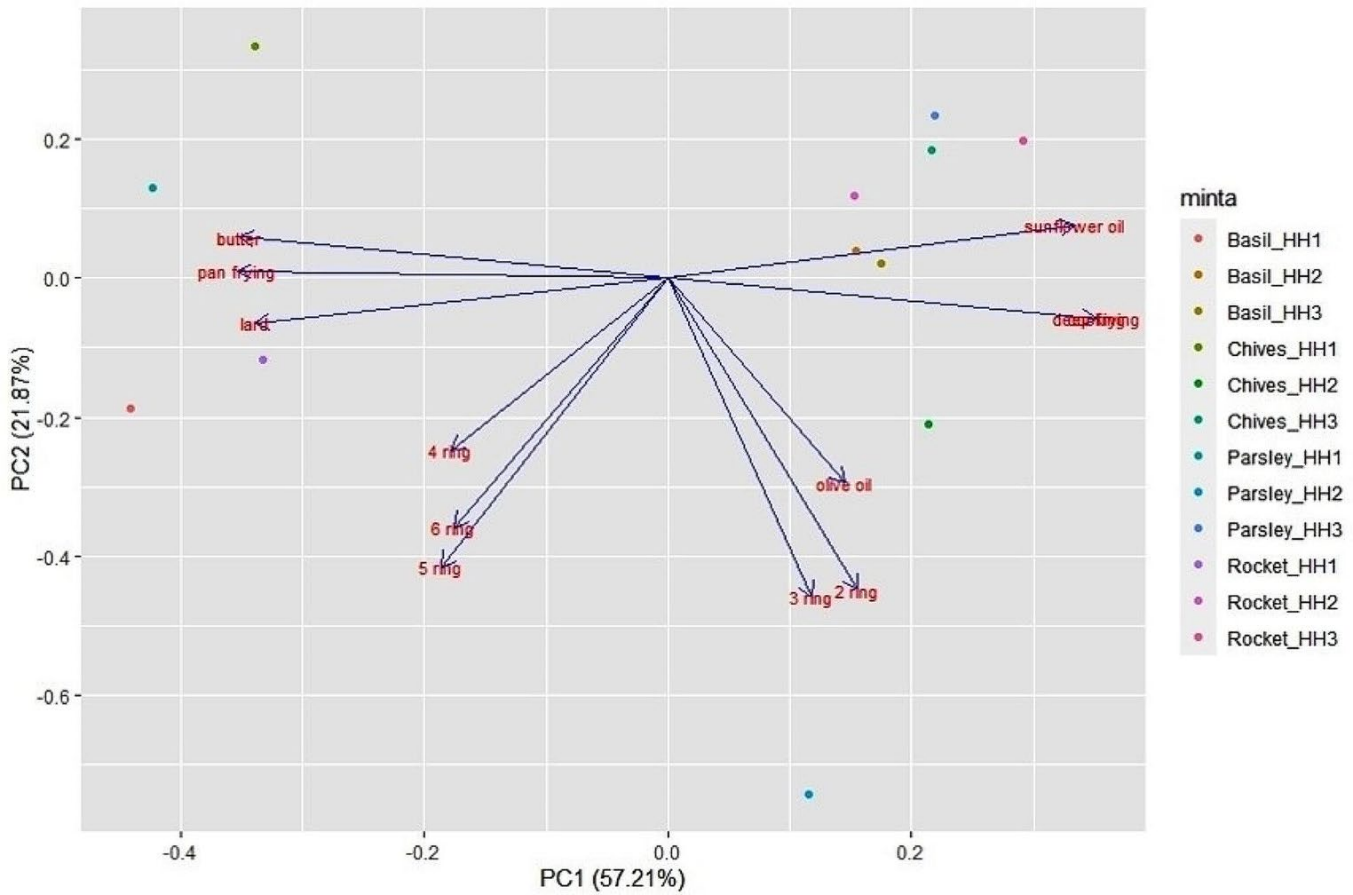
PCA diagram of the samples based on PAHs isomers and cooking habits/materials used

PCA has been a widely used tool to identify possible emission sources of PAHs [22, 48]. In our study, PCA is based on the PAHs content of different vegetables and the cooking behaviour of the three households. It explains 79.01% of the variance in the data in a single two-dimensional model (Fig. 2). Four groups were determined, high correlation was found between (I) frequency of using olive oil, 2 ring and 3 ring PAHs, (II) frequency of using butter or lard and pan frying; (III) 4, 5, 6 ring PAHs; (IV) frequency of using sunflower oil, deep frying and baking in oven.

Olive oil usage seems to be associated with the emission of 2- and 3-ring PAHs. It was used in only one household (HH2) at relatively high frequency (app. 5%). Chiang et al. [10] measured total, gaseous-phase and particle-phase PAHs emission rates when different vegetable oils (soybean oil, palm oil, and olive oil) were used for deep-frying. Of gaseous-phase PAHs, naphthalene amounted to 87% in olive oil. Comparing the three different oils, palm oil emitted significantly higher particle-phase PAHs than the other two.

HMW (4–6 ring) PAHs form another group, without clear relationship with potential sources. In general, frying operations such as stir- and deep-frying have been associated with the production of higher molecular weight compounds [46, 60]. These operations are reported for all three households being present in the study.

### Potential health hazards

Concerning carcinogenicity of PAHs, different classification systems exist (reviewed by Sampaio et al. [44]. 13 PAHs were classified as genotoxic and carcinogenic by the Joint FAO/WHO Expert Committee on Food Additives: B(a)A, Chr, CPP (Cyclopenta[cd]pyrene), B(b)F, B(a)P, 5-MC (5-Methylchrysene), B(j)F (Benzo[j]fluoranthene), B(k)F, DBA, Ind, D(e)P (Dibenzo[a,e]pyrene), D(i)P (Dibenzo[a,i]pyrene), and dibenzo[a,h]pyrene. Of them, four PAHs (B(a)A, Chr, B(b)F, and B(a)P) were identified as occurring in food and being good indicators of toxicity (EFSA 2008).

B(b)F was detected in all samples, reaching the highest values (above 13 µg/kg) in basil and rocket samples of HH1 (13.8 µg/kg and 13.2 µg/kg, respectively) and in the parsley sample of HH1 (13.5 µg/kg) (Table 1). B(a)A also occurred in every sample, with the highest concentrations in all HH2 samples: 9.8 µg/kg in basil, 11.9 µg/kg in rocket, 9.0 µg/kg in parsley and 8.5 µg/kg in chives (Table 1). Cry was also detected in every vegetable sample, reaching outstandingly high concentration, 20.1 µg/kg, in the rocket sample in HH2.

Finally, B(a)P occurred in 5 samples, in the range of 1.6 µg/kg (chives, HH1) and 5.7 µg/kg (parsley, HH1). B(a)P is ubiquitous in heat-treated foods [45]. Yao et al. [60] found B(a)P emission characteristic during deep frying. Hu et al. [19] measured concentrations of 8 PAHs including B(a)P in sunflower oil during deep-frying under simulated frying conditions and demonstrated an increase with increasing frying time.

## Conclusions

Accumulation of PAHs was assessed in 3 Hungarian kitchens using common culinary herbs over 1 month exposure. Households participating in the study were of similar size and they were located in villages not affected by heavy traffic but they were representing different cooking practices, using different cooking oils. PAHs profiles in the test plants could be associated to cooking habits of these households, resulting in characteristic differences between the households in the distribution of HMW PAHs. In HH2, concentration of 6-ring PAHs was 11.1 µg/kg in basil and 7.8 µg/kg in parsley. Similarities were also detected, especially in case of LMW PAHs: Naphthalene was almost exclusively the dominant PAH in the tested herbs, with concentrations varying between 25.4 µg/kg and 274 µg/kg. Phenanthrene was the dominant 3-ring PAH, in the majority of the samples, reaching as much as 62 µg/kg in parsley in HH1. Culinary herbs used in our study are often recommended for cultivation and use in kitchens. However, many of the test plants accumulated carcinogenic PAHs such as benzo[a]anthracene (highest concentration 11.9 µg/kg in rocket in HH2), benzo[b]fluoranthene (highest concentration 13.8 µg/kg in basil in HH2 but very similar concentrations, 13.5 µg/kg in parsley in HH1 and 13.2 µg/kg in rocket also in HH2), as well as chrysene (highest concentration 20.1 µg/kg in rocket in HH2) which might question their safe use.

## Data Availability

All data generated or analysed during this study are included in this published article.

## Abbreviations

PM: particulate matter
PAH: polycyclic aromatic hydrocarbon
LMW: low molecular weight
HMW: high molecular weight
Naphthalene: Nap
Acenaphthylene: Acy
Acenaphthene: Ace; Fluorene– Flu
Phenanthrene: Phen
Anthracene: Ant
Fluoranthene: Flt
Pyrene: Pyr
Benzo[a]anthracene: B(a)A
Chrysene: Cry
Benzo[b]fluoranthene: B(b)F
Benzo[k]fluoranthene: B(k)F
Benzo[e]pyrene: B(e)P
Benzo[a]pyrene: B(a)P
Indeno[1,2,3-cd]pyrene: Ind
Dibenzo[a,h]anthracene: D(a,h)a
Benzo[g,h,i]perylene: B(g,h,i.)p
